# Pediatrics

**Published:** 1895-05

**Authors:** Maud J. Frye


### PEDIATRICS.

Reported by MAUD J. FRYE, M. D.

### PYREXIA IN ACUTE DISEASES OF CHILDHOOD.

IN THE March number of *Archives of Pediatrics* Dr. C. G. Jennings, of Detroit, considers the treatment of pyrexia in the acute diseases of childhood. Aconite, the bath and the synthetic antipyretics are the remedies discussed. The method of administration and indications for use of each of these remedies are so well set forth that a considerable extract from the article is here given.

Aconite is best given for the control of pyrexia, after the method of Ringer—to a child of three years of age from one-sixth to one-quarter of a drop of the tincture should be given every fifteen minutes for the first hour and every hour after that for ten or twelve doses; then the cardiac sedation may be maintained by the same dose every two, three or four hours.

The most generally useful method of applying water is by the full tub bath. For the treatment of pyrexia in general, a bath, beginning at a temperature of 95° and gradually reduced by adding cold water or ice until the temperature of the water is 85°, is the most generally useful. Children endure cold badly and they should be carefully guarded against the fright that a sudden immersion in cold water is almost sure to bring. The duration of the bath should be from five to ten minutes. During the continuation of it the cutaneous capillary circulation should be excited by vigorous rubbing. After the bath the child should be dried and placed in dry sheets. The application of an ice bag or cold water to the head during the bath is advisable. Should the child fail to react promptly, or shiver from the effect of the application, a small dose of hot whisky may be given to aid reaction.

The writer has used each of the three drugs, acetanilid, antipyrin and phenacetin, exclusively for many months in succession. Phenacetin has given, by far, the most satisfactory results. Clinicians generally agree that powerful effects, like cyanosis and cardiac depression, almost never occur with this drug in reasonable dosage.

The beginning dose of phenacetin should never be larger than one-half grain to each year of age. This dose may be repeated every hour for two or three doses. Guided by the thermometer or the relief of the distress and the breaking out of a gentle perspiration, the administration of the drug can be arrested, when the result is accomplished. The action of aconite upon the heart and circulation indicates its use in the beginning of all the acute diseases attended by local engorgements. Acute nasal and pharyngeal catarrh, tonsilitis, laryngitis, bronchitis, pneumonia and pleurisy, in their first stages, are particularly amenable to this drug; but antipyresis and the relief of the unpleasant symptoms of fever in these conditions comes with aconite alone but slowly or not at all. Much better results can be obtained in all of the conditions by combining with the aconite one, two or three proper doses of phenacetin at hourly intervals at the height of the pyrexia. After the period of engorgement in pneumonia, or any grave inflammatory disease, aconite should have no place in its therapeutics and antipyresis should be accomplished by the bath or phenacetin, administered with very great care. Whenever there is doubt as to the strength of the heart the bath should be selected.

In the pyrexia resulting from the absorption of ptomaines and the acute indigestions of children, phenacetin is, *par excellence*, the remedy. It can be administered with calomel, bismuth and salol to suit the necessity of the case. In the pyrexia of acute intestinal catarrh, entero-colitis and cholera infantum the coal tar antipyretics or aconite should not be used. These conditions, attended as they are by vaso-motor paresis, are most scientifically treated by the bath. In cholera infantum this is the most powerful remedy we have to restore vaso-motor tone and reduce hyperpyrexia.

In the acute inflammations of the cerebral and spinal meninges phenacetin will be the antipyretic to administer. The cutaneous hyperesthesia and general irritability of these children preclude the disturbance attending the use of the bath and, furthermore, the coal tar antipyretics are powerful nerve sedatives. In meningitis and cerebro-spinal meningitis they promptly relieve the agonizing headache and neuralgic pains and induce quiet and refreshing sleep.

In the exanthematous fevers aconite is sometimes of value to depress the excited circulation in the beginning, but it is a drug that should be administered in these conditions with extreme caution. In measles the high fever attending the onset and acme of the eruptive stage can be well and safely controlled by phenacetin. In the high temperature and nervous disturbance and the internal congestion that attends the delayed appearance of the rash, the bath is by far the preferable measure.

In scarletina the cool bath should be one of the therapeutic measures in all cases. In cases of mild and moderately severe type, with a temperature from 102° to 103°, the administration of one or two doses of phenacetin, when the pyrexia becomes uncomfortable, is good treatment. In severe cases, the temperature rising to 104° or above, the bath is the remedy. Occasional administration of one or two doses of phenacetin, to prolong its antipyretic action, may be of value. In grave cases with a suppressed rash, high temperature and grave nervous phenomena the bath vigorously used will relieve symptoms and save life.

---

It is claimed that, at least, ten per cent. of the patients at the chief dispensary of the City of New York suffer from tea-drunkenness. Its effects are noticed in connection with the nervous system.—*Maryland Medical Journal.*

